# A Novel Forkhead Box Protein P (FoxP) From *Litopenaeus vannamei* Plays a Positive Role in Immune Response

**DOI:** 10.3389/fimmu.2020.593987

**Published:** 2020-12-14

**Authors:** Jiefeng Gao, Ran Geng, Hengwei Deng, Hongliang Zuo, Shaoping Weng, Jianguo He, Xiaopeng Xu

**Affiliations:** ^1^ State Key Laboratory of Biocontrol, School of Life Sciences, Sun Yat-sen University, Guangzhou, China; ^2^ Southern Marine Science and Engineering Guangdong Laboratory (Zhuhai), Zhuhai, China; ^3^ Institute of Aquatic Economic Animals and Guangdong Provice Key Laboratory for Aquatic Economic Animals, Sun Yat-sen University, Guangzhou, China

**Keywords:** Forkhead box protein P, invertebrate, *Litopenaeus vannamei*, immune response, humoral immunity, phagocytosis, immune regulation

## Abstract

The forkhead box protein P (FoxP) family members have been known to be important for regulation of immune responses in vertebrates, but their roles in invertebrate immunity remain unclear. In this study, a novel FoxP gene (LvFoxP) was identified from Pacific white shrimp *Lito*p*enaeus vannamei* and functionally studied in the context of immune response. Possessing a conserved FoxP coiled-coil domain and a forkhead domain, LvFoxP shared homology to vertebrate FoxP family members, in particular FoxP1. Expression of LvFoxP was detectable in all the examined tissues and could be up-regulated by immune challenge in gill and hemocytes. The LvFoxP protein was present in both the cytoplasm and nucleus of hemocytes and could be nuclear-translocated upon immune stimulation. Silencing of LvFoxP increased the susceptibility of shrimp to infections by *Vibrio parahaemolyticus* and white spot syndrome virus (WSSV) and down-regulated the expression of multiple components of NF-κB and JAK-STAT pathways and almost all the examined immune effector genes. Moreover, the phagocytic activity of hemocytes from LvFoxP-silenced shrimp against *V. parahaemolyticus* was decreased. These suggested that LvFoxP could play a positive role in immune response. The current study may provide novel insights into the immunity of invertebrates and the functional evolution of the FoxP family.

## Introduction

The Forkhead box (Fox) protein superfamily consists of a large group of transcriptional regulators with a forkhead/winged-helix DNA-binding domain, which are essentially implicated in regulation of development, homeostasis and metabolism (1, 2). In human, based on sequence homology, Fox proteins are further categorized into 19 subfamilies (Fox A to FoxS) (3). The FoxP family consists of four members, FoxP1 to FoxP4, the evolutionary origin of which can be traced back to sea lampreys and invertebrates (4–6). Mammalian FoxP family members generally function as transcriptional repressors or activators involving in tumor suppression, development and, in particular, immunity (5). For instance, FoxP3 plays critically roles in development and function of regulatory T cells (Tregs) by regulating expression of many genes and is identified as a classic marker specifically for CD4^+^CD25^+^ Tregs (7). By attenuating the efficacy of protective immune responses, the CD4^+^CD25^+^ FoxP3^+^ Treg cells alleviate tissue damages caused by infection and inflammation to benefit the host in various circumstances (8, 9). FoxP1 and FoxP4 are also well known to be involved in development and function of lymphatic cells (10, 11). At present, more and more sequences of the FoxP family member genes from various invertebrates have been deposited in Genbank database. Previous studies have showed that *Drosophila* FoxP is important for motor coordination and operant learning, and may be functionally homologous to vertebrate FoxP2 (12, 13). However, roles of the FoxP family in invertebrate immunity have not been explored so far.

Pacific white shrimp *Lito*p*enaeus vannamei*, the representative species of crustaceans, is the major cultured shrimp species with the highest yield in the world (14). Because of its important economical values and evolutionary status, the *L. vannamei* immune system has attracted more and more research attentions (15, 16). Accumulating studies have proved fruitful in systematically characterizing the nature and regulatory mechanisms of the immune response in *L. vannamei*, which makes *L. vannamei* an important model for studying invertebrate immunity. In the current study, a novel FoxP gene (LvFoxP) was identified from *L. vannamei* and functionally studied in the context of immune response. We demonstrated that LvFoxP was implicated in regulation of humoral and cellular immunity in shrimp, playing important roles in defensing against *Vibrio parahaemolyticus* and white spot syndrome virus (WSSV) infections. To our knowledge, this is the first study exploring the function of an invertebrate FoxP gene in immunity, which may provide novel insights into the immune system of crustaceans and help to learn more about the functional evolution of the FoxP family.

## Materials and Methods

### Shrimp and Pathogens

Pacific white shrimp (~10 g) were obtained from an aquaculture farm in Zhuhai, Guangdong Province, which had been randomly sampled to ensure free of *Vibrio parahaemolyticus* and WSSV by PCR following previously described methods (17, 18). Shrimp were acclimated at ~27°C for at least 7 days in a recirculating water tank system filled with air-pumped seawater (2.0% salinity). The *V. parahaemolyticus*, *Staphylococcus aureus*, and WSSV isolated from aquacultured shrimp were kept in our lab (19–21).

### Bioinformatics Analysis

The coding sequence of LvFoxP retrieved from the reported *L. vannamei* genome and transcriptome data (22) was verified by RT-PCR. The full length of LvFox mRNA was determined by rapid amplification of cDNA ends (RACE) using a SMARTer™ RACE cDNA Amplification kit (Clontech, Japan) according to the manufacturer’s protocol. Sequence alignments between LvFoxP and other FoxP family members were analyzed by Clustal W 1.8. using the Gonnet series matrix with a gap extension penalty of 0.2, a gap opening penalty of 10, and a delay of divergent sequences of 30%. The phylogenetic tree was generated with MEGA 5.0 software by the neighbor-joining (NJ) method using a Poisson model with parameters as follows: substitution type (amino acid), rates among sites (uniform rates), gap missing deletion treatment (complete deletion), and number of bootstrap replication (1000 replicates).

### Real-Time PCR

For analysis of the distribution of LvFoxP mRNA, various tissues were sampled and pooled from 15 individual healthy shrimp. For analysis of LvFoxP transcription during immune responses, *L. vannamei* were intramuscularly injected with 50 µl PBS (as control) or PBS containing *V. parahaemolyticus* (10^6^ CFU), *S. aureus* (10^5^ CFU), and WSSV (10^6^ copies). The gill and hemocytes were sampled at 0, 4, 12, 24, 48, 72, and 96 h post injection. Total RNA was purified using a RNeasy Plus Mini Kit (Qiagen, Germany), and cDNA was reverse transcribed using a PrimeScript RT reagent kit (Takara, Japan) according to the manufacturer’s instructions. Each sample was analyzed using real-time PCR in quadruplicate on a LightCycle 480 System (Roche, Germany). The 10 μL real-time PCR amplification system contained 1 μL cDNA, 5 μL 2×SYBR Premix Ex Taq™ II (Takara, Japan), and 500 nM of each primer. The optimized thermal cycling parameters were 95°C for 30 s, followed by 40 cycles of 95°C for 5 s, 60°C for 30 s, and 78°C for 1 s. The expression levels of genes were normalized to those of the internal control gene elongation factor 1 alpha (EF1-α, Genbank accession no. GU136229). Experiments were repeated in triplicate and sequences of primers used were listed in **Table S1**.

### Confocal *L*aser Scanning Microscopy

The coding sequence of LvFoxP was cloned into the pAc5.1-GFP vector (23) and transfected into *Drosophila* S2 cells at a confluence of ~80% using FuGENE HD Transfection (Promega, USA) to express green fluorescent protein (GFP)-tagged LvFoxP protein. At 24 h later, cells were stained with Hoechst 33342 (Sigma, USA) and visualized under a confocal laser scanning microscope (Leica TCS-SP5, Germany).

### Immunofluorescence

*L. vannamei* were challenged with *V. parahaemolyticus* (10^6^ CFU), WSSV (10^6^ copies), poly (I:C) (10 µg), lipopolysaccharide (LPS, 10 µg) and PBS (as control), and at 24 h later, hemolymph smear samples were made on siliconized slides, fixed with 4% paraformaldehyde for 10 min, and treated with 1% Triton X-100 for 20 min. After blocking with 10% normal goat serum, slides were incubated with rabbit antibody specific to the N-terminal region of LvFoxP (customized and purchased from GL Biochem, China), followed by Alexa fluor 488 conjugated goat anti-rabbit IgG antibody (Abcam, USA). Before observation, slides were stained with Hoechst 33342 (Sigma, USA) for the nuclei.

### Western Blot

*L. vannamei* were immune challenged as described above, and at 24 h later, total nuclear and cytoplasmic protein of hemocytes pooled from 30 *L. vannamei* were extracted using NE-PER Nucler and Cytoplasmic Extraction Reagents (Thermo, USA). Samples were detected by western-blot using rabbit anti-LvFoxP antibody (GL Biochem, China). The anti-Histone H3 (CST, USA) and anti-β-actin antibodies (MBL, Japan) were used for detections of the nuclear and cytoplasmic internal controls, respectively. The gray values of the LvFoxP specific band were calculated using the Quantity One 4.6.2 software (Bio-Rad, USA) by Gauss model using three different rolling disk sizes (5, 10, and 15) and normalized to that of Histone H3 or β-actin as previously described (24).

### RNA Interference (RNAi)

Double stranded RNAs (dsRNAs) specific to LvFoxP (dsLvFoxP) and GFP (dsGFP, as control) were synthesized by *in vitro* transcription with a T7 RiboMAX™ Express RNAi System (Promega, USA) according to the manufacturer’s protocol. Each *L. vannamei* was injected with 1 μg/1 g body weight dsLvFoxP and dsGFP. Gills from nine shrimp in each group were sampled and pooled at 48 h post injection. The efficiency of LvFoxP silencing and expression of a series of immune related genes were analyzed using real-time PCR. Also at 48 h post injection, dsRNA-treated shrimp were further challenged with 10^6^ copies of WSSV or 10^6^ CFU of *V. parahaemolyticus* (*n* = 50 in each group). Experiments were done in triplicate and the cumulative mortality was recorded. The bacterial load of *V. parahaemolyticus* in hepatopancreas pooled from nine shrimp at 48h post infection (hpi) in each group was determined by a conventional plate count method on TCBS agar plates. The copy number of WSSV in muscle sampled at 48 and 72h post infection (hpi) was analyzed by quantitative real-time PCR. Briefly, the primers WSSV32678-qRTF and WSSV32753-qRTR and the Taqman probe WSSV32706 were used to amplify the DNA extracted from muscle tissues. The standard curve was generated by amplification of a 10-fold gradient dilution of a pMD19-T plasmid (Takara, Japan) containing the corresponding WSSV genomic DNA fragment. The WSSV genome copy numbers in 1 g of muscle tissue were then calculated. Sequences of the primers and Taqman probe were listed in **Table S1**.

### Phagocytic Activity Analysis

Hemocytes from *L. vannamei* at 48 h post dsRNA injection were washed with 2× Leibovitz’s L-15 medium (Gibco, USA) and mixed with latex beads (Merck, Germany), fluorescein isothiocyanate (FITC)-labled *V. parahaemolyticus* or *S. aureus* at a 1:100 ratio of cells/particles. After incubation at 28°C for 1 h, hemocytes were detected using Accuri C6 flow cytometer (BD, USA) for the signals of FITC and the forward scatter (FSC) values of cell size. The threshold of FSC was determined by detection of free latex beads, FITC-labeled *V. parahaemolyticus* or *S. aureus*, and the fluorescence boundary was set based on detection of the self-fluorescence of untreated hemocytes (**Figure S1**).

### Dual Luciferase Reporter Assays

The coding sequence of LvFoxP was cloned into pAc5.1/V5-His A plasmid (Invitrogen, USA) to generate LvFoxP expression vector. The previously reported firefly luciferase plasmid PGL-Basic vectors (Promega, USA) containing promoters of antimicrobial peptides (AMPs), Dorsal, Relish, SWDs, CTL4 and lysozyme were used (25–29). *Drosophila* S2 cells were plated in 96-well plate and transfected with 0.05 μg firefly luciferase plasmid, 0.1 μg gene expression vector, and 0.03 μg pRL-TK renilla luciferase plasmid (as internal control) (Promega, USA). Cells were harvested at 48 h post transfection and lysed for examination of firefly and Renilla luciferase activities using a Dual-Luciferase Reporter Assay System (Promega, USA). Three independent experiments were performed, and all assays were performed in eight independent transfections.

### Statistical Analysis

Statistical comparisons were performed by two-tailed unpaired Student’s t test or one-way ANOVA followed by Dunnett’s *post hoc* test using SPSS software. The Log-rank (Mantel-Cox) test was used to analyze the survival rates. Data are presented as mean ± standard deviation (SD).

## Results

### Sequence Analysis of LvFoxP

**Figure 1:**
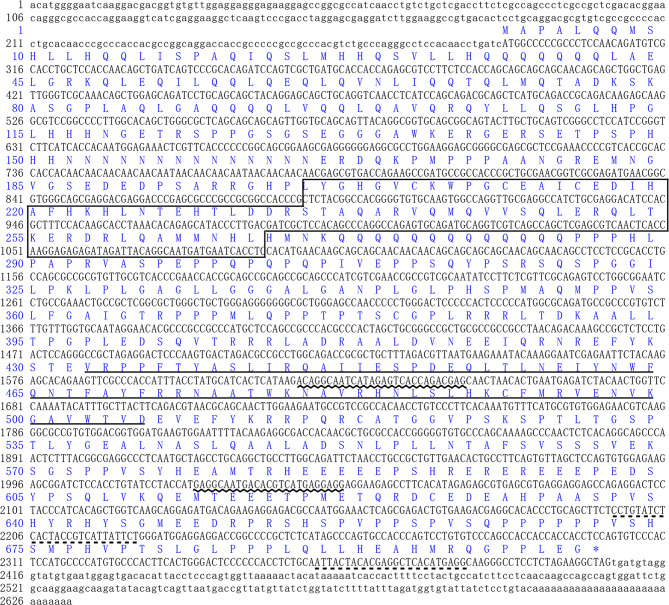
Sequence analyses of *L. vannamei* LvFoxP. The sequences of ORF and 5’-/3’-UTR were shown with upper and lower cases, respectively. The deduced amino acid was represented with one-letter code above the nucleotide sequence. The FoxP coiled-coil and the forkhead domains were boxed and underlined, respectively. The positions of primers for dsRNA amplification and real-time PCR were underlined with wavy and dotted lines, respectively.

**Figure 2:**
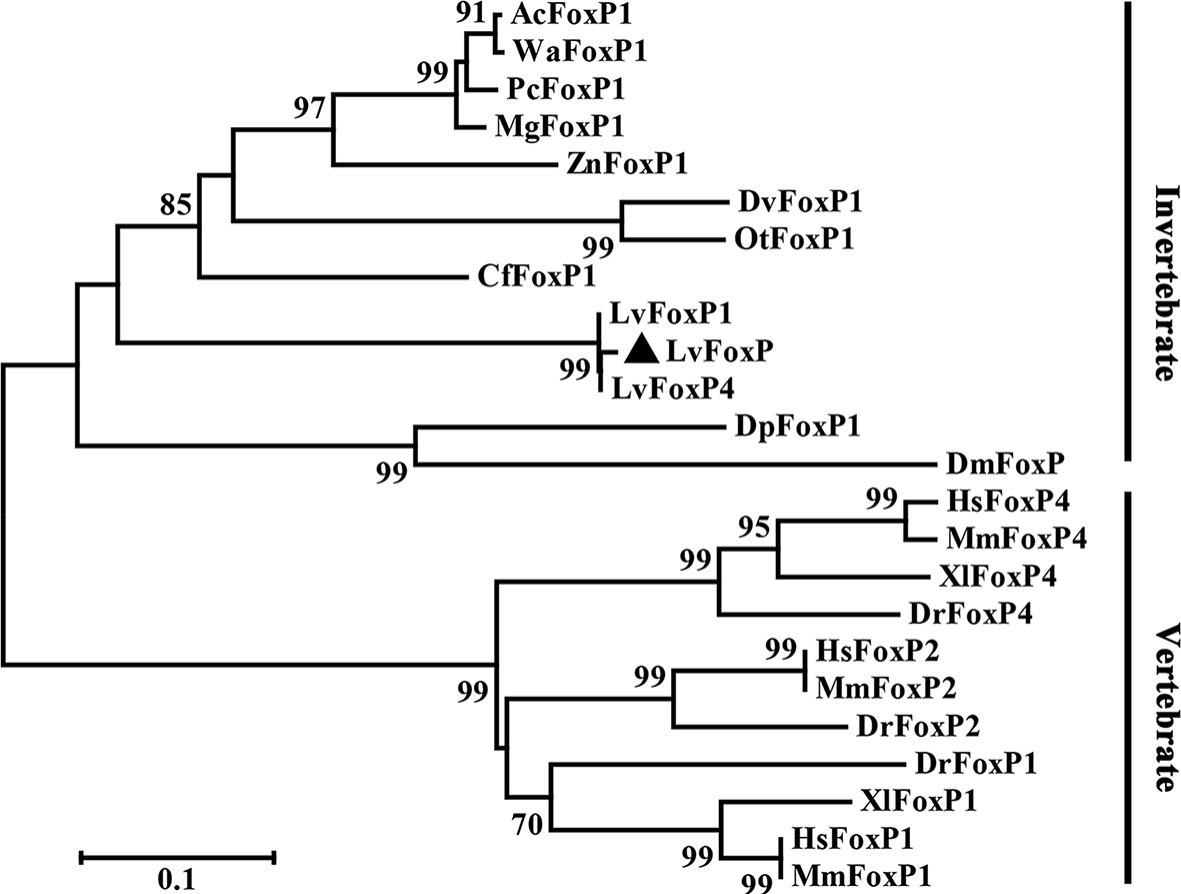
Neighbor joining phylogenetic tree analysis of the amino acid sequences of the analyzed FoxP family members from vertebrates and invertebrates. LvFoxP was marked with a black triangle. Proteins analyzed included: LvFoxP1 from *Litopenaeus vannamei* (Genbank accession no. XP_027221045.1); LvFoxP4 from *Litopenaeus vannamei* (ROT70546.1); AcFoxP1 from *Atta cephalotes* (XP_012055440.1); WaFoxP1 from *Wasmannia auropunctata* (XP_011693623.1); PcFoxP1 from *Polistes canadensis* (XP_014606511.1); MgFoxP1 from *Megalopta genalis* (XP_033323454.1); ZnFoxP1 from *Zootermopsis nevadensis* (XP_021940550.1); DvFoxP1 from *Diabrotica virgifera* (XP_028138981.1); OtFoxP1 from *Onthophagus taurus* (XP_022901118.1); CfFoxP1 from *Ctenocephalides felis* (Genbank accession XP_026472380.1); DpFoxP1 from *Drosophila pseudoobscura* (XP_033241608.1); DmFoxP from *Drosophila melanogaster* (NP_001247011.1); DrFoxP1 from *Danio rerio* (XP_005162017.1); DrFoxP2 from *Danio rerio* (NP_001025253.1); DrFoxP4 from *Danio rerio* (NP_001186420.1); XlFoxP1 from *Xenopus laevis* (Q5W1J5.1); XlFoxP4 from *Xenopus laevis* (Q4VYR7.1); HsFoxP1 from *Homo sapiens* (NP_001336266.2); HsFoxP2 from *Homo sapiens* (NP_001166237.1); HsFoxP4 from *Homo sapiens* (NP_001012426.1); MmFoxP1 from *Mus musculus* (NP_001184251.1); MmFoxP2 from *Mus musculus* (NP_001273536.1); MmFoxP4 from *Mus musculus* (XP_017173175.1).

The full length of LvFoxP mRNA is 2632 bp with a 288-bp 5’ untranslated region (UTR) and a 226-bp 3’ UTR. The open reading frame (ORF) of LvFoxP is 2118 bp encoding a 705-amino acid protein with a calculated molecular weight of 78.6 kDa and a theoretical isoelectric point of 6.65 (**Figure 1**). At present, a few FoxP family members from invertebrates have been deposited on the Genbank database, although their sequences have not been experimentally verified. Sequence comparison was performed between LvFoxP and a series of FoxPs, including some of these putative invertebrate FoxPs, by multiple-sequence alignment. Although the sequence similarities between LvFoxP and the analyzed vertebrate FoxP3 were low (22–27%) (**Figure S2**), LvFoxP showed high homologies with other FoxP family members (**Figure S3**). The sequence similarities between LvFoxP and the putative LvFoxP1 and LvFoxP4 from *L. vannamei*, DpFoxP1 from *Drosophila pseudoobscura*, DmFoxP from *Drosophila melanogaster* were 72, 46, 32, and 33%, respectively. In contrast, LvFoxP shared similarities of 44, 44, and 43% with FoxP1 and 35, 35, and 36% with FoxP4 from human, mouse and zebrafish, respectively. The similarities between LvFoxP and FoxP2 from these vertebrate species were all 39%. Notably, in the analyzed sequences, the FoxP coiled-coil domain and especially the forkhead domain were much higher conserved than other regions. The four putative DNA-binding sites in the Forkhead domain were almost identical in these sequences. The constructed phylogenetic tree showed that the analyzed FoxP family proteins could be clustered into invertebrate and vertebrate categories, and LvFoxP was classified into the invertebrate group and most close to the putative LvFoxP1 and LvFoxP4 (**Figure 2**).

### Subcellular Localization and Tissue Distribution of LvFoxP

**Figure 3:**
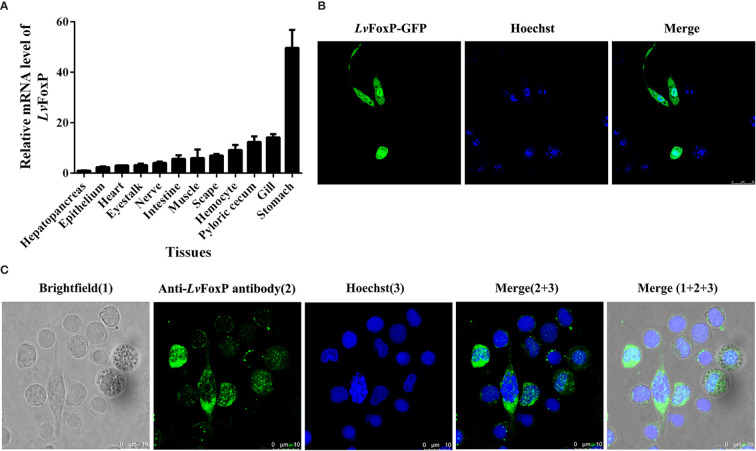
Subcellular and tissue distribution of LvFoxP. **(A)** Distribution of LvFoxP mRNA in *L. vannamei* tissues. Data were presented as fold values relative to the lowest level in hepatopancreas (set as 1.0). Each bar represents the mean ± SD of three detections. **(B)** Subcellular localization of GFP-tagged LvFoxP (green) in *Drosophila* S2 cells observed by confocal laser scanning microscopy. **(C)** Immunofluorescent analysis of LvFoxP expression in hemocytes using specific antibody. The nucleus was stained with Hoechst 33342 (blue).

The distribution of LvFoxP mRNA in tissues was analyzed using real-time PCR (**Figure 3A**). Stomach expressed the highest level of LvFoxP, which was 49.7 times higher than the lowest level in hepatopancreas. Expression of LvFoxP in gill, pyloric cecum (a digestive organ), hemocyte, and scape (the first segment of antenna) was 14.2-, 12.4-, 9.2-, and 7.0-fold that in hepatopancreas. Other tissues expressed moderate levels of LvFoxP. Confocal laser scanning microscopy demonstrated that the GFP-tagged LvFoxP was present in both cytoplasm and nucleus in S2 cells (**Figure 3B**). The result was convinced by immunofluorescence using an anti-LvFoxP antibody, the specific of which was verified by western-blot analysis of hemocytes and S2 cells expressing the GFP-tagged LvFoxP (**Figure S4**). The LvFoxP protein could be detected in the cytoplasm and nucleus of hemocytes from unstimulated shrimp (**Figure 3C**).

### Activation of LvFoxP Upon Immune Stimulation

**Figure 4:**
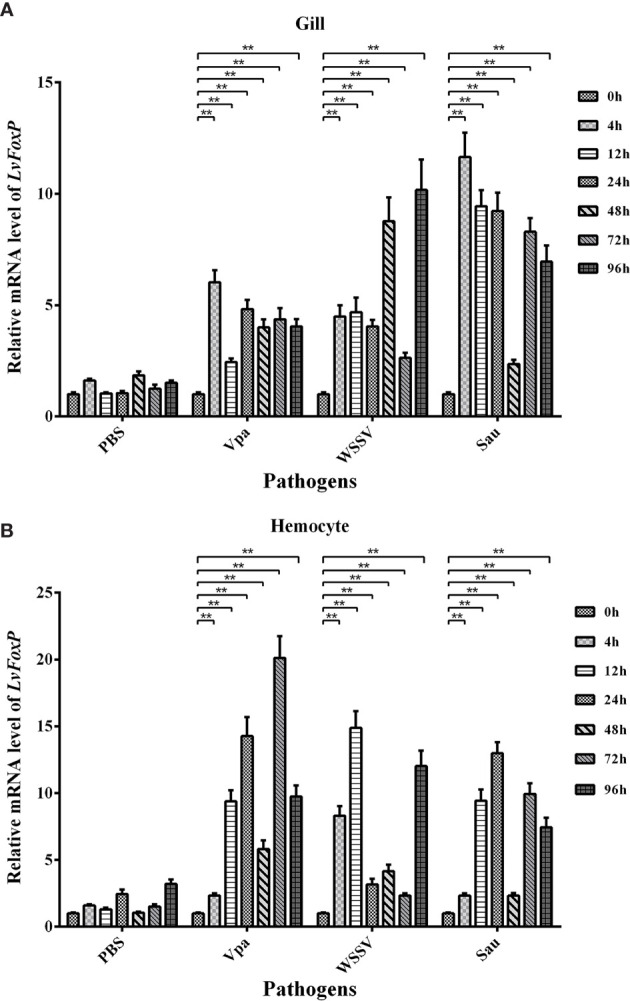
Expression profiles of LvFoxP *in vivo* after immune stimulations. Expression of LvFoxP in gill **(A)** and hemocytes **(B)** from shrimp infected with *V. parahemolyticus* (Vpa), *S. aureus* (Sau), and WSSV was analyzed by real-time PCR with the EF-1α gene as the internal control. Data are representative of three experiments and presented as means ± SD of four parallel detections. **P < 0.01 by one way ANOVA with Dunnett’s *post hoc* test comparing to the control (0 h, set as 1.0).

**Figure 5:**
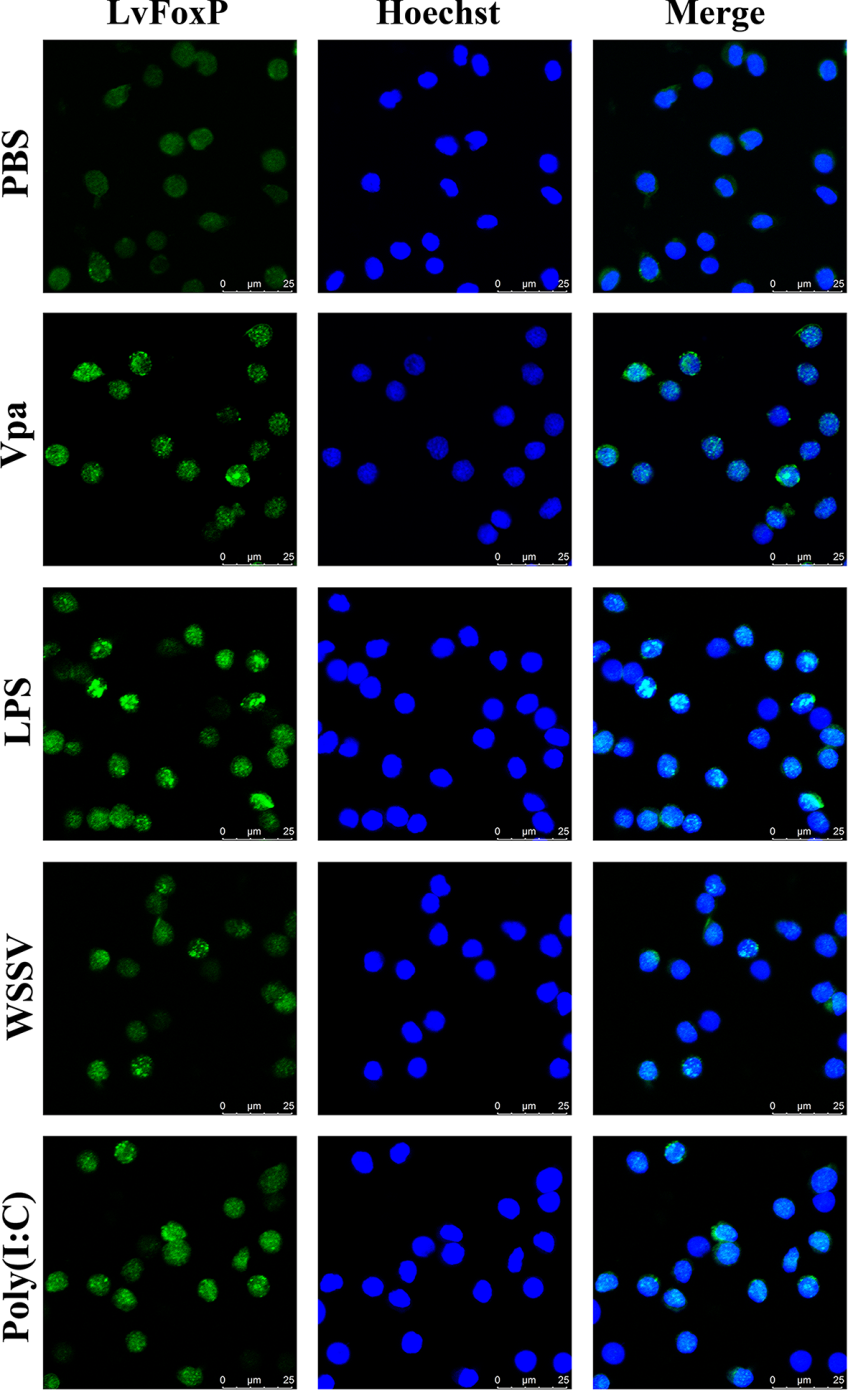
Nuclear translocation of LvFoxP upon immune stimulations analyzed by immunofluorescence. Hemocytes were extracted from *L. vannamei* challenged with *V. parahaemolyticus* (Vpa), LPS, WSSV, Poly (I:C) and PBS (as control). Results are representative of three experiments.

**Figure 6:**
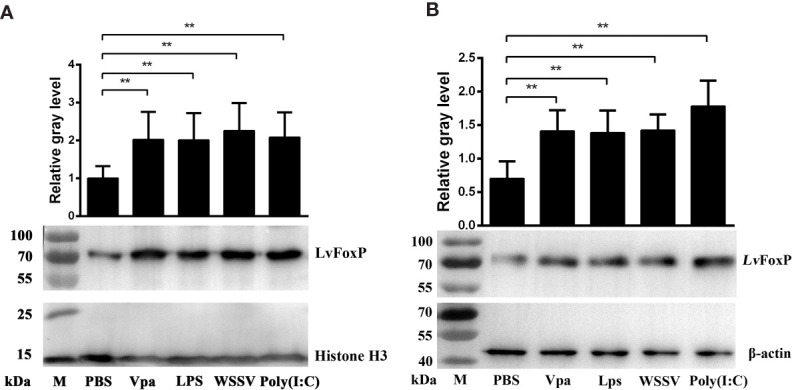
Western-blot analysis of nuclear translocation of LvFoxP upon immune stimulations. The LvFoxP protein in the nuclear **(A)** and cytoplasmic **(B)** extracts of hemocytes from immune-challenged shrimp was analyzed. The gray values of LvFoxP bands were normalized to those of the nuclear internal control Histone H3 or the cytoplasmic internal control β-actin. Results are representative of three experiments. **P < 0.01 by one way ANOVA with Dunnett’s *post hoc* test.

The expression profiles of LvFoxP after immune stimulations with bacterial and viral pathogens were investigated using real-time PCR. In both gill and hemocytes, the mRNA level of LvFoxP was significantly increased after *V. parahaemolyticus*, *S. aureus* and WSSV infections and the rising of LvFoxP expression exhibited periodic trends (**Figure 4**). *V. parahaemolyticus* and WSSV are the two major pathogens most harmful to shrimp. We then investigated their effects on cellular localization of LvFoxP. The immunofluorescent assay demonstrated that compared with the PBS control, both *V. parahaemolyticus* and WSSV and their simulated stimulants LPS and ploy (I:C) efficiently promoted the nuclear translocation of LvFoxP in hemocytes (**Figure 5**). The results were further confirmed by western-blot, which showed that compared with the control, the protein level of LvFoxP was increased in the nucleus of hemocytes from *V. parahaemolyticus-*, WSSV-, LPS-, and ploy (I:C)-stimulated shrimp (**Figure 6A**). Concurrently, the level of LvFoxP in the cytoplasm was also increased in the cytoplasm (**Figure 6B**), which further confirmed that expression of LvFoxP could be up-regulated by immune challenges.

### Roles of LvFoxP in Immune Responses

**Figure 7:**
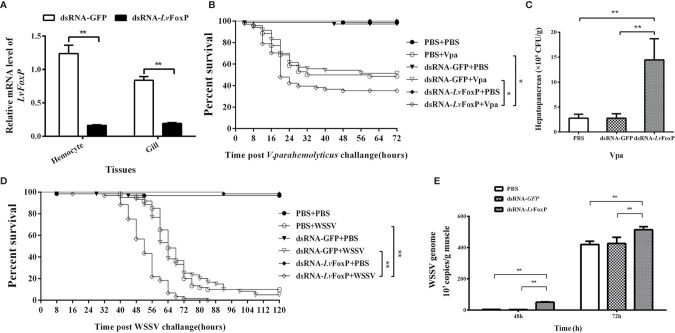
Roles of LvFoxP in immune responses. **(A)** Real-time PCR analysis of the silencing efficiency of LvFoxP in gill at 48 h post dsRNA injection. **(B**, **D)** Survival rates of LvFoxP-, GFP-dsRNA and PBS (as control) treated *L. vannamei* after infection with *V. parahemolyticus* and WSSV. Data are representative of three independent experiments which showed similar results and were statistically analyzed using Log-rank (Mantel-Cox) test (*p < 0.05). **(C)** The bacterial load of *V. parahemolyticus* in hepatopancreas analyzed by plate count method. **(E)** The viral load of WSSV in muscle analyzed by absolute quantitative real-time PCR. Data are representative of three experiments and each bar represents the mean ± SD of four detections. **P < 0.01 by two-tailed unpaired Student’s t test.

The expression of LvFoxP in shrimp was silenced by injection with dsRNA based on RNAi strategy (**Figure 7A**). Shrimp were further challenged with WSSV and *V. parahaemolyticus*. Compared with the dsGFP treated control, the survival rates of LvFoxP-silenced shrimp were significantly decreased after *V. parahaemolyticus* infection (**Figure 7B**). The final survival rates of dsLvFoxP- and dsGFP-treated shrimp were 34.3 and 51.4% after *V. parahaemolyticus* infection, respectively. The bacterial load of *V. parahaemolyticus* in hepatopancreas was also increased by 5.2-fold after treatment with dsLvFoxP (**Figure 7C**). Although there is little difference between the final survival rates of experimental and control groups, the death time of shrimp was significantly advanced after silencing of LvFoxP (**Figure 7D**). The copy number of WSSV in muscle from dsLvFoxP treated shrimp was increased by 14.7-fold at 48 h post infection (hpi), although it was only slightly increased at 72 hpi (**Figure 7E**). These suggested that LvFoxP could play positive roles in both antiviral and antibacterial responses.

### Regulation of Phagocytosis by LvFoxP

**Figure 8:**
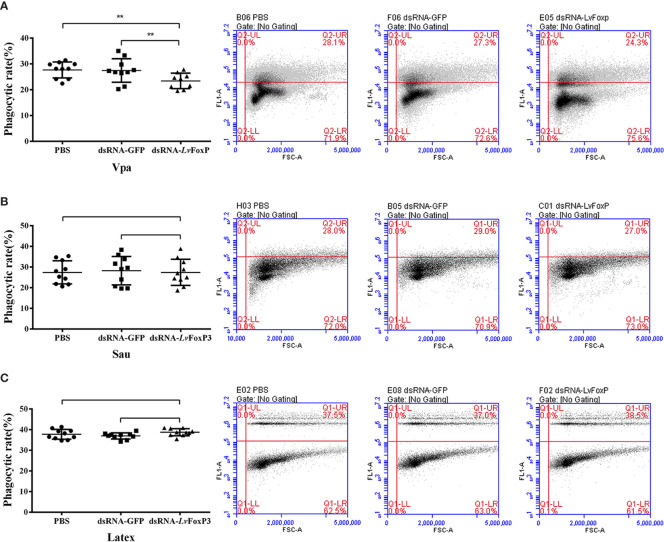
Regulation of phagocytosis by LvFoxP. The phagocytic activity of hemocytes extracted from dsLvFoxP, dsGFP and PBS (as control) treated shrimp against FITC-labeled *V. parahaemolyticus* (Vpa) **(A)**, *S. aureus* (Sau) **(B)** and latex beads **(C)** were analyzed using flow cytometry. In the left panels, each dot represents the test result of one shrimp. In the right panels, the scatter plots represent one of the three detections. Cells were examined by forward scatter (FSC, x-axis) for size and by intracellular green fluorescence (y-axis) for phagocytized latex beads or FITC-labeled bacteria. **P < 0.01 by two-tailed unpaired Student’s t test.

The phagocytic activity of hemocytes is a key part of the cellular immunity for fighting against bacterial infection in shrimp. We then investigated the effects of LvFoxP on hemocyte phagocytosis using flow cytometry. Compared with the control, the phagocytic activity of hemocytes from LvFoxP-silenced shrimp against *V. parahaemolyticus* was decreased (**Figure 8A**), while those against *S. aureus* and latex beads did not change (**Figures 8B, C**). These suggested that LvFoxP may mainly promote hemocyte phagocytosis of *V. parahaemolyticus*.

### Regulation of Immune-Related Genes by LvFoxP

**Figure 9:**
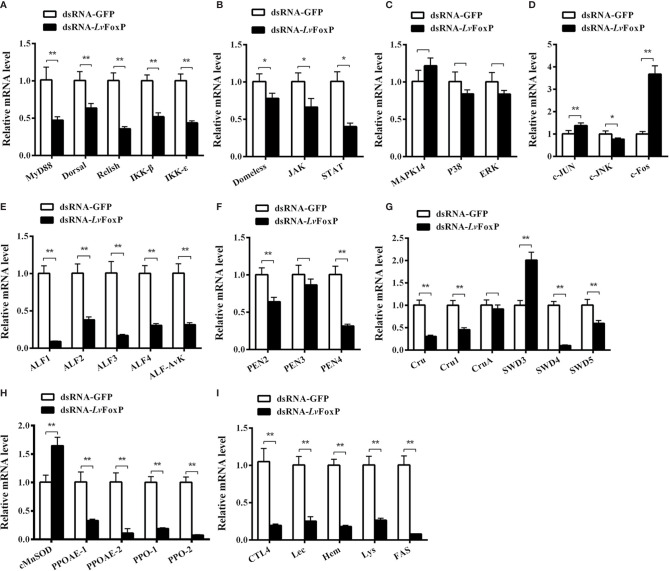
Regulatory effects of LvFoxP on expression of immune related genes. Expression of LvFoxP *in vivo* was silenced using RNAi strategy. The regulatory effects of LvFoxP-silencing on expression of the components of the NF-κB (Dorsal and Relish) pathway **(A)**, JAK-STAT pathway **(B)**, p38 MAPK and ERK pathways **(C)** and JNK pathway **(D)**, and immune functional genes including antimicrobial peptides ALFs **(E)**, PENs **(F)**, SWDs **(G)**, and immune functional proteins cMnSOD and prophenoloxidases (PPO) **(H)**, and hemolin (Hem), lysozyme (Lys), and fatty acid synthase (FAS) and C-type lectins CTL4 and Lec **(I)** in gills were investigated using real-time PCR. Results are representative of three independent experiments with data presented as means ± SD of four detections. *P < 0.05 and **P < 0.01 by two-tailed unpaired Student’s t test comparing to the dsGFP treated sample (set as 1.0).

To further explore the role of LvFoxP in immunity, expression of a series of immune related genes after silencing of LvFoxP *in vivo* was investigated using real-time PCR. Silencing of LvFoxP significantly down-regulated expression of the NF-κB pathway components Dorsal, Relish, Myd88, IKKβ, and IKKϵ, and the JAK-STAT pathway components Domeless, JAK and STAT, indicating that LvFoxP could promote activation of these signaling pathways (**Figures 9A, B**). In contrast, compared with the control, expression of the MAPK family kinases p38 MAPK, MAPK14, ERK, and c-JNK, and down-stream transcription factor c-Jun did not change significantly in LvFoxP-silenced shrimp, while only that of the transcription factor c-Fos was up-regulated (**Figures 9C, D**). These suggested that LvFoxP may not affect the activity of most MAPK pathways but may inhibit activation of the c-Fos-mediated pathways. Furthermore, upon silencing of LvFoxP, expression of almost all the examined immune effector genes involved in humoral immunity, including AMPs, such as anti-lipopolysaccharide factor (ALF) 1-4 and -Avk, penaeidin (PEN) 2 and 4, Crutin (Cru) and Cru1, single whey acidic protein domain-containing peptide (SWD) 4 and 5, and immune functional proteins such as prophenoloxidase (PPO)-1 and -2, PPO-activating enzyme (PPOAE)-1 and -2, C-type lectins CTL4 and Lec, the immunoglobulin superfamily member hemolin (Hem), lysozyme (Lys), and fatty acid synthase (FAS), was significantly down-regulated, while only that of SWD3 and cytosolic manganese superoxide dismutase (cMnSOD) was increased (**Figures 9E–I**). Only the expression of PEN3 and CruA was not significantly changed after LvFoxP silencing. These indicated that LvFoxP could be positively involved in regulation of shrimp humoral immunity.

**Figure 10:**
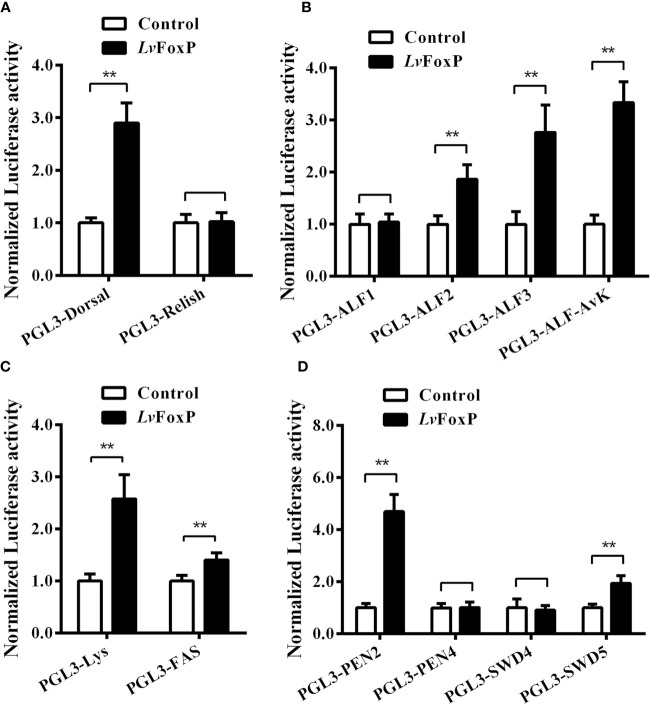
Regulatory effects of LvFoxP on promoters of many immune related genes. Dual-luciferase reporter assays were performed on PGL3 vectors containing promoters of Dorsal and Relish **(A)**, ALFs **(B)**, lysozyme (Lys) and fatty acid synthase (FAS) **(C)**, and PENs and SWD5 **(D)**. Data are means ± SD (*n* = 8). **P < 0.01 by one way ANOVA with Dunnett’s *post hoc* test.

**Figure 11:**
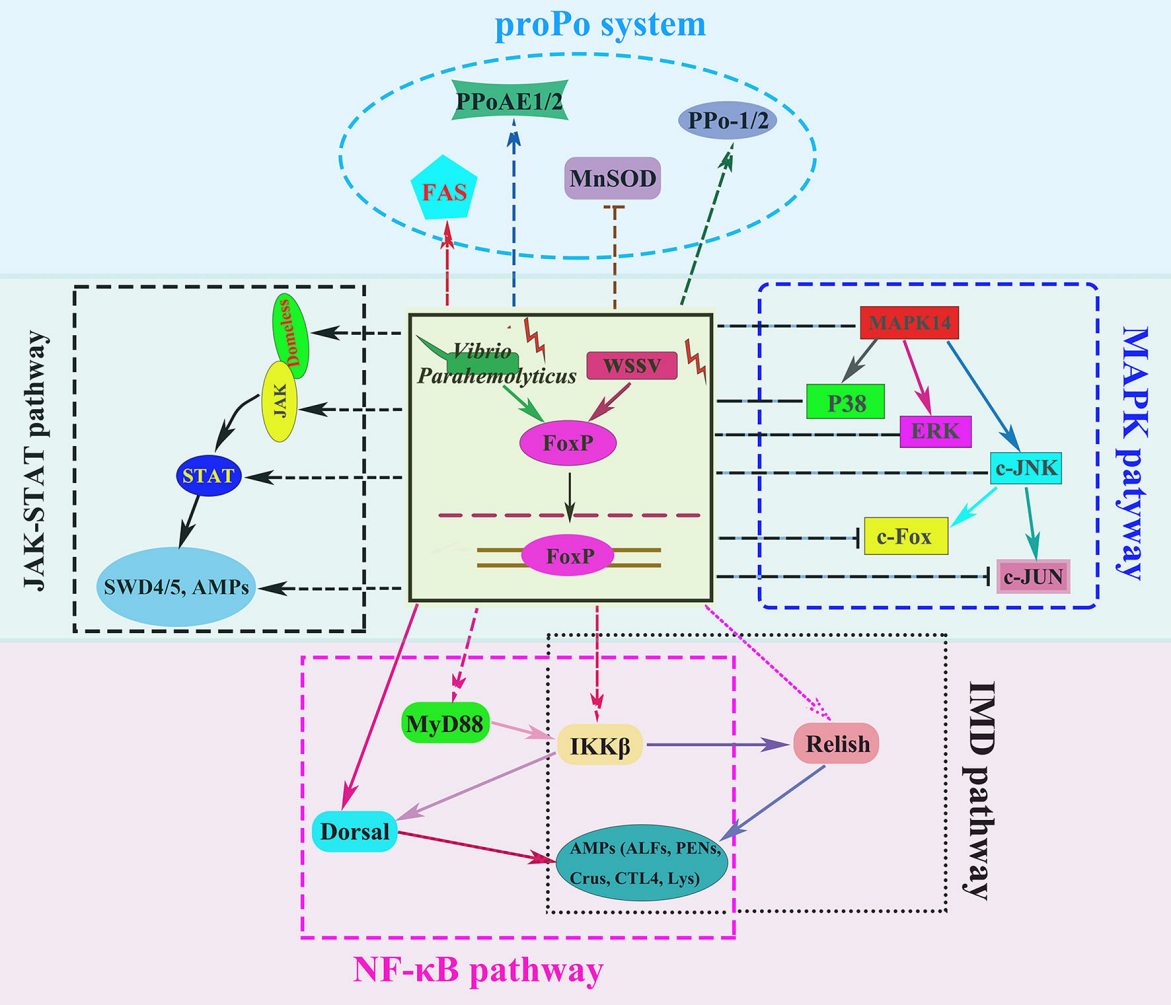
Diagram of the regulatory effects of LvFoxP on various signaling pathways and immune effector genes in shrimp. The arrows and T-shaped lines were used to represent activation and inhibition effects, respectively, and the solid and dotted lines to represent direct and indirect (or unknown) effects, respectively.

To further investigate the regulation of immune related genes by LvFoxP, the effects of LvFoxP on promoters of Dorsal, Relish and 10 immune effector genes were investigated using dual-luciferase reporter assays (**Figure 10**). The results demonstrated that expression of LvFoxP enhanced activities of the promoters of Dorsal, ALF2, ALF3, ALF-AVK, PEN2, SWD5, Lys, and FAS. However, LvFoxP showed no effects on promoters of the Relish, ALF1, PEN4, and SWD4 genes, the expression of which had been shown to be decreased by silencing of LvFoxP *in vivo*. These suggested that LvFoxP may regulate their expression by indirect ways. Furthermore, the regulatory effects of LvFoxP on signaling pathways and immune effector genes in shrimp analyzed in this study were diagrammatically summarized in **Figure 11**.

## Discussion

To date, dozens of FoxP proteins from invertebrates, in particular insects and crustaceans, have been deposited in the Genbank database, indicating that FoxP could be ubiquitously present in invertebrate species. However, unlike their homologues in mammals, most invertebrate FoxPs have not been functionally studied. The current study identified and functionally studied a novel FoxP family member LvFoxP in shrimp. The expression of LvFoxP was activated after immune challenges with bacteria and virus, and the LvFoxP protein could be translocated into the nucleus upon immune stimulation. As nuclear translocation is a sign of the activation of the FoxP family members (30, 31), these suggested that the function of LvFoxP could be closely related to immune response. Silencing of LvFoxP decreased expression of most immune effector proteins analyzed in this study, including CTLs, lysozyme, hemolin, PPOs, and AMPs from ALF, PEN, and Crustin families, which are all essential for humoral immune responses. Previous studies have described that PENs, ALFs and SWDs, and secreted immune functional proteins hemolin and CTLs possess both antibacterial and antiviral activities in shrimp (25, 28, 32, 33). It can be inferred that through up-regulating expression of these proteins, LvFoxP may enhance immune responses in shrimp. This was confirmed by the pathogen challenging experiment, which demonstrated that knockdown of LvFoxP *in vivo* increased the susceptibilities of shrimp to infections by *V. parahaemolyticus* and WSSV.

The mechanisms underlying the regulatory effects of LvFoxP on expression of immune related genes are worthy of further investigation. However, although silencing of LvFoxP widely decreased expression of the analyzed genes, dual-luciferase reporter assays showed that LvFoxP could only regulate the promoters of a few of them. These indicated that LvFoxP may regulate gene expression by either direct or indirect ways. Some genes, the promoters of which were not directly targeted by LvFoxP, may be regulated by indirect ways. It has been known that the regulation of immune responses in both vertebrate and invertebrate animals is largely governed by the NF-κB, MAPK, and JAK-STAT signaling pathways (21, 23, 34). Upon silencing of LvFoxP, expression of the detected components of shrimp NF-κB and JAK-STAT pathways was significantly down-regulated, suggesting that LvFoxP could exert positive effects on activation of these two pathways. The only two members of the NF-κB family in shrimp, Dorsal and Relish, are essential for expression of a series of AMPs and CTLs, many of which possess both antibacterial and antiviral activities (27, 28, 35). The JAK-STAT pathway in shrimp also controls expression of a series of AMPs that are implicated in immune responses against *V. parahaemolyticus* and WSSV (36, 37). These suggested that LvFoxP may regulate expression of immune functional proteins for promotion of humoral immunity by acting directly or through these pathways.

As an important organ of digestive system, shrimp stomach expresses high level of LvFoxP, suggesting a potential role of LvFoxP in digestion. Since the stomach may directly encounter microorganisms invading through ingestion, this also indicates that LvFoxP may play a role in the anti-microbial immune response in the stomach, which requires in-depth exploration. The LvFoxP protein was also high expressed in hemocytes and could be translocated into the nucleus upon immune stimulation, indicating that LvFoxP may play a role related to the immune function of hemocytes. Latex beads are common biomaterials widely used in detection of cell phagocytosis (38). The current work showed that silencing of LvFoxP in shrimp did not affect hemocyte phagocytosis of latex beads and *S. aureus* but reduced that of *V. parahaemolyticus*. These indicated that the mechanism underlying hemocyte phagocytosis of *V. parahaemolyticus* could be different from those of other foreign particles, which requires further exploration. Previous studies have suggested that many secreted immune functional proteins, including these detected in this study, such as SWD4, SWD5, hemolin, and CTLs, could enhance hemocyte phagocytosis of bacteria in arthropods (25, 33, 39, 40). The opsonization mechanisms mediated by these LvFoxP-regulated proteins could contribute to the stimulatory effect of LvFoxP on hemocyte phagocytosis. Taken together, the enhancement of LvFoxP to shrimp immune responses against *V. parahaemolyticus* could be a synergistic action of its regulatory effects on humoral and cellular immunities, which is worthy of further studies.

The current study concerned the role of a FoxP family member in invertebrate immunity, which may be of significance for exploring the functional evolution of FoxPs. The novel identified LvFoxP shared high homology with putative LvFoxP1 and LvFoxP4 from the same species that have not yet been experimentally verified. Moreover, LvFoxP was highly homologous to FoxP1 from mammals and other vertebrate species, suggesting that there may be an evolutionary relationship between LvFoxP and FoxP1. Previous data have shown that mammalian FoxP1, FoxP2, and FoxP4 share high homology in sequence (41, 42). LvFoxP was also homologous with these two mammalian FoxP family members, especially in the forkhead domain region. So, we think it may be difficult to determine the evolutionary status of LvFoxP simply from its sequence. At present, the researches on FoxP2 mainly focus on its role in language and brain development in human (43), while functions of both FoxP1 and FoxP4 have been well known to be closely related to the immune system (11, 44). Foxp1 is essential for the development and function of T cell-independent B-1 B cells that are essential for innate immunity (10, 45). However, as previously described, Foxp1 also plays a negative role in differentiation of follicular helper T cells and suppresses expression of many immune-related genes, indicating a possible repressor role of FoxP1 in immune responses (46, 47). In contrast, FoxP4 is required for T cells to produce effector cytokines in response to antigen stimulation, although it is not dispensable for development of T cells (9). These suggested the complex functions of the FoxP family in vertebrates. The current work demonstrated that LvFoxP played positive roles in both humoral and cellular immunity in shrimp, also indicating that the function of LvFoxP may not be directly analogized to its homologous genes in mammals. Therefore, we think the functional evolutionary relationship between LvFoxP and the vertebrate FoxP family requires further exploration.

## Data Availability Statement

The datasets presented in this study can be found in online repositories. The names of the repository/repositories and accession number(s) can be found in the article/**Supplementary Material**.

## Author Contributions

XX and JH supervised the overall project and designed the experiments. XX wrote the manuscript. JG and RG performed the experiments and analyzed data with the help from HD, HZ, and SW. All authors contributed to the article and approved the submitted version.

## Funding

This work was funded by the National Natural Science Foundation of China under grant nos. 31772881, 31702371, 31972823, and 32073004; the National Key Research and Development Program of China 2018YFD0900505; the Natural Science Foundation of Guangdong Province, China 2017A030313190; the China Agriculture Research System CARS47; and The Fundamental Research Funds for the Central Universities of China 18lgpy57.

## Conflict of Interest

The authors declare that the research was conducted in the absence of any commercial or financial relationships that could be construed as a potential conflict of interest.

## Supplementary Material

Supplementary Figure 1 Setting of the thresholds of FSC and fluorescence signals for the flow cytometry analysis of hemocyte phagocytosis. The fluorescence boundary was set based on detection of the self-fluorescence of untreated hemocytes (left pannels), and the threshold of FSC was determined by detection of free latex beads, FITC-labeled *V. parahaemolyticus* (Vpa) or *S. aureus* (Sau) (right pannels).

Supplementary Figure 2 Multiple-sequence alignment of LvFoxP with vertebrate FoxP3. The identical amino acid residues were shaded in black and the similar residues in gray. The FoxP coiled-coil domain and forkhead domain regions were marked with arrows, and the high conserved residues in the forkhead region were boxed. The FoxP3 proteins analyzed included: HsFoxP3 from *Homo sapiens* (Genbank accession no. NP_054728.2); MmFoxP3 from *Mus musculus* (NP_001186276.1); DrFoxP3 from *Danio rerio* (ACQ44666.1); CcFoxP3 from *Cyprinus carpio* (XP_018923635.1); XlFoxP3 from *Xenopus laevis* (NP_001121199.1); NpFoxP3 from *Nanorana parkeri* (XP_018413763.1); RbFoxP3 from *Rhinatrema bivittatum* (XP_029463377.1).

Supplementary Figure 3 Multiple-sequence alignment of LvFoxP with other FoxP family members. The identical amino acid residues were shaded in black and the similar residues in gray. The FoxP coiled-coil domain and forkhead domain regions were marked with arrows, and the high conserved residues in the forkhead region were boxed. The FoxPs analyzed included: LvFoxP1 from *Litopenaeus vannamei* (Genbank accession no. XP_027221045.1); LvFoxP4 from *Litopenaeus vannamei* (ROT70546.1); DpFoxP1 from *Drosophila pseudoobscura* (XP_033241608.1); DmFoxP from *Drosophila melanogaster* (NP_001247011.1); DrFoxP1 from *Danio rerio* (XP_005162017.1); DrFoxP2 from *Danio rerio* (NP_001025253.1); DrFoxP4 from *Danio rerio* (NP_001186420.1); HsFoxP1 from *Homo sapiens* (NP_001336266.2); HsFoxP2 from *Homo sapiens* (NP_001166237.1); HsFoxP4 from *Homo sapiens* (NP_001012426.1); MmFoxP1 from *Mus musculus* (NP_001184251.1); MmFoxP2 from *Mus musculus* (NP_001273536.1); MmFoxP4 from *Mus musculus* (XP_017173175.1).

Supplementary Figure 4 Analysis of the specificity of the anti-LvFoxP antibody used in this study. Left panel: western-blot analysis of S2 cells expressing GFP (as control, line 1) and GFP-tagged LvFoxP (line 2) using anti-GFP antibody (Invitrogen, USA). Right panel: western-blot analysis of of S2 cells expressing GFP (line 1), GFP-tagged LvFoxP (line 2), and shrimp hemocytes (line 3, expressing natural LvFoxP) using anti-LvFoxP antibody.
