# Supplementary figures and images for: A Novel Forkhead Box Protein P (FoxP) From Litopenaeus vannamei Plays a Positive Role in Immune Response
Source: Front Immunol. 2020 Dec 14;11:593987. doi: 10.3389/fimmu.2020.593987 (PMC7768020; doi:10.3389/fimmu.2020.593987)

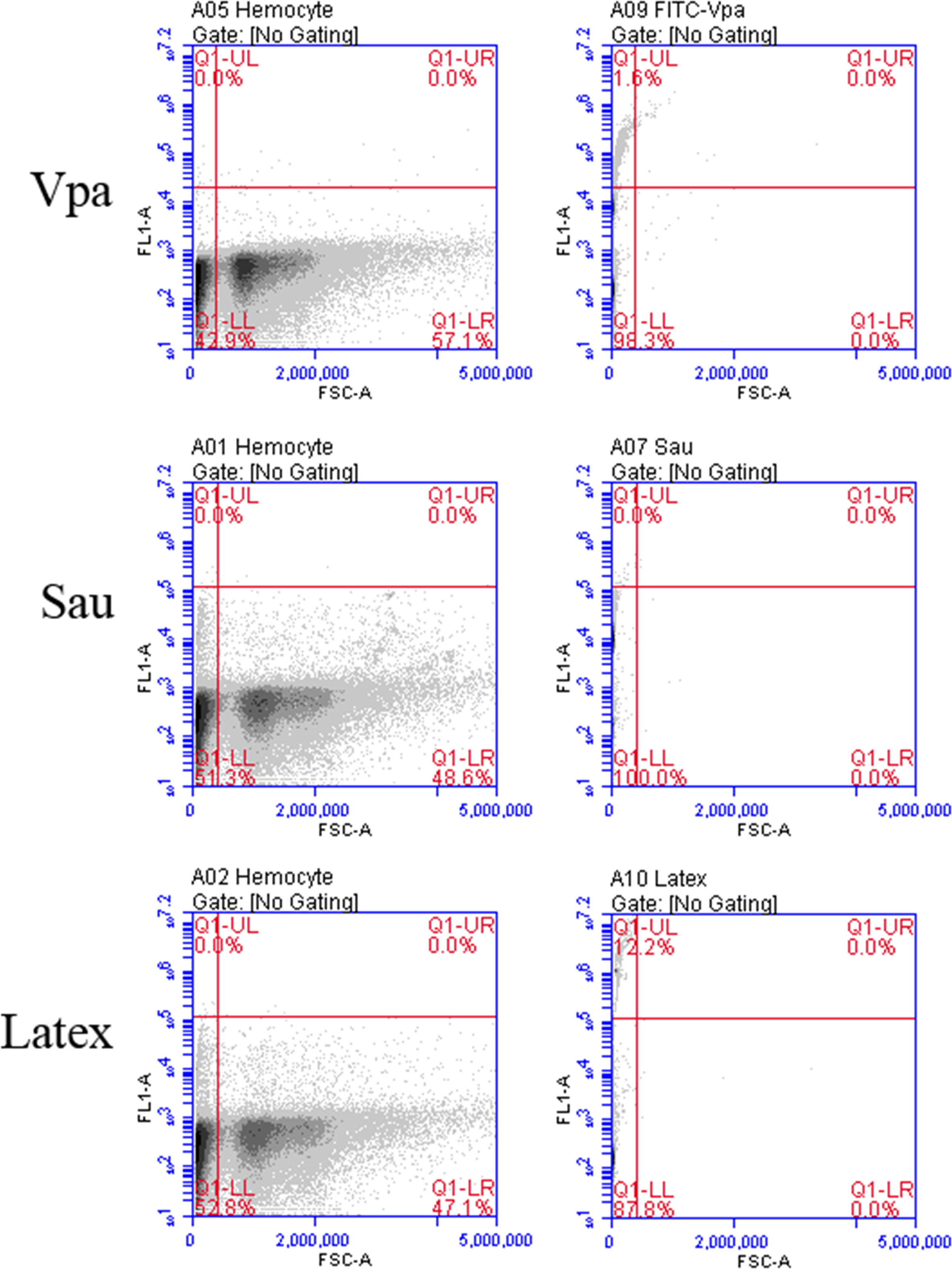

Supplement: Supplementary Figure 1 — Setting of the thresholds of FSC and fluorescence signals for the flow cytometry analysis of hemocyte phagocytosis. The fluorescence boundary was set based on detection of the self-fluorescence of untreated hemocytes (left pannels), and the threshold of FSC was determined by detection of free latex beads, FITC-labeled V. parahaemolyticus (Vpa) or S. aureus (Sau) (right pannels). [file Image_1.tif]

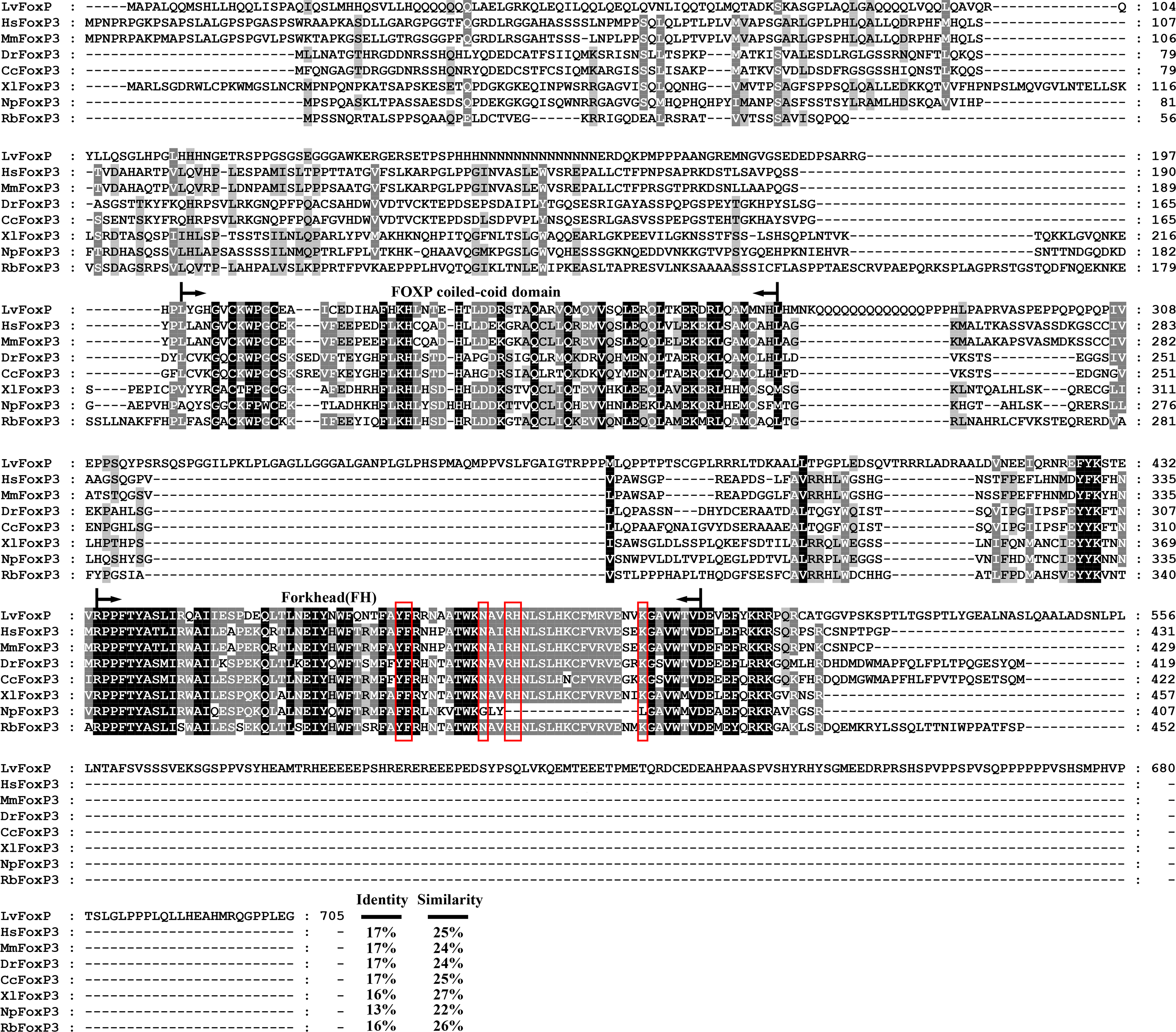

Supplement: Supplementary Figure 2 — Multiple-sequence alignment of LvFoxP with vertebrate FoxP3. The identical amino acid residues were shaded in black and the similar residues in gray. The FoxP coiled-coil domain and forkhead domain regions were marked with arrows, and the high conserved residues in the forkhead region were boxed. The FoxP3 proteins analyzed included: HsFoxP3 from Homo sapiens (Genbank accession no. NP_054728.2); MmFoxP3 from Mus musculus (NP_001186276.1); DrFoxP3 from Danio rerio (ACQ44666.1); CcFoxP3 from Cyprinus carpio (XP_018923635.1); XlFoxP3 from Xenopus laevis (NP_001121199.1); NpFoxP3 from Nanorana parkeri (XP_018413763.1); RbFoxP3 from Rhinatrema bivittatum (XP_029463377.1). [file Image_2.tif]

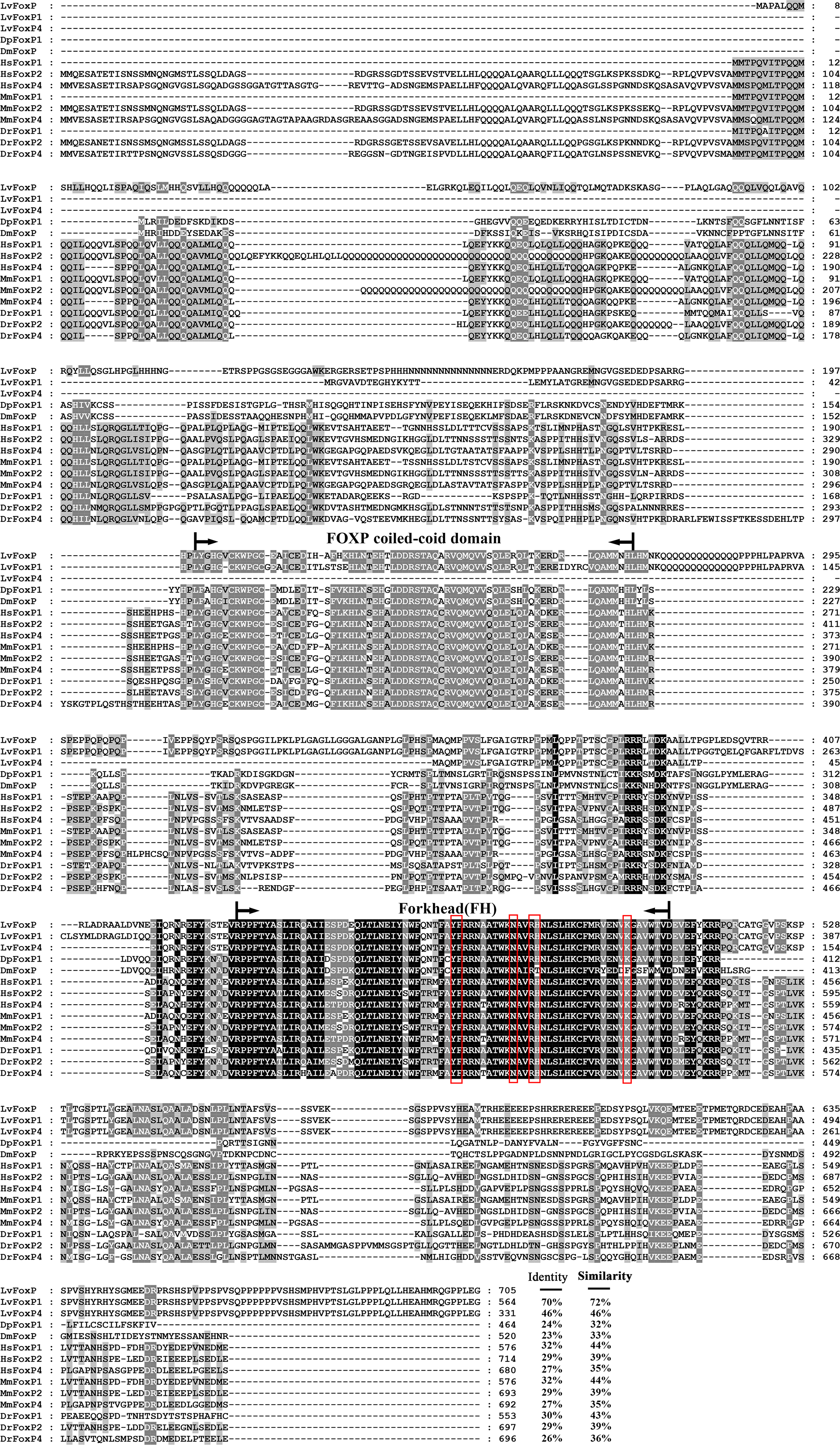

Supplement: Supplementary Figure 3 — Multiple-sequence alignment of LvFoxP with other FoxP family members. The identical amino acid residues were shaded in black and the similar residues in gray. The FoxP coiled-coil domain and forkhead domain regions were marked with arrows, and the high conserved residues in the forkhead region were boxed. The FoxPs analyzed included: LvFoxP1 from Litopenaeus vannamei (Genbank accession no. XP_027221045.1); LvFoxP4 from Litopenaeus vannamei (ROT70546.1); DpFoxP1 from Drosophila pseudoobscura (XP_033241608.1); DmFoxP from Drosophila melanogaster (NP_001247011.1); DrFoxP1 from Danio rerio (XP_005162017.1); DrFoxP2 from Danio rerio (NP_001025253.1); DrFoxP4 from Danio rerio (NP_001186420.1); HsFoxP1 from Homo sapiens (NP_001336266.2); HsFoxP2 from Homo sapiens (NP_001166237.1); HsFoxP4 from Homo sapiens (NP_001012426.1); MmFoxP1 from Mus musculus (NP_001184251.1); MmFoxP2 from Mus musculus (NP_001273536.1); MmFoxP4 from Mus musculus (XP_017173175.1). [file Image_3.tif]

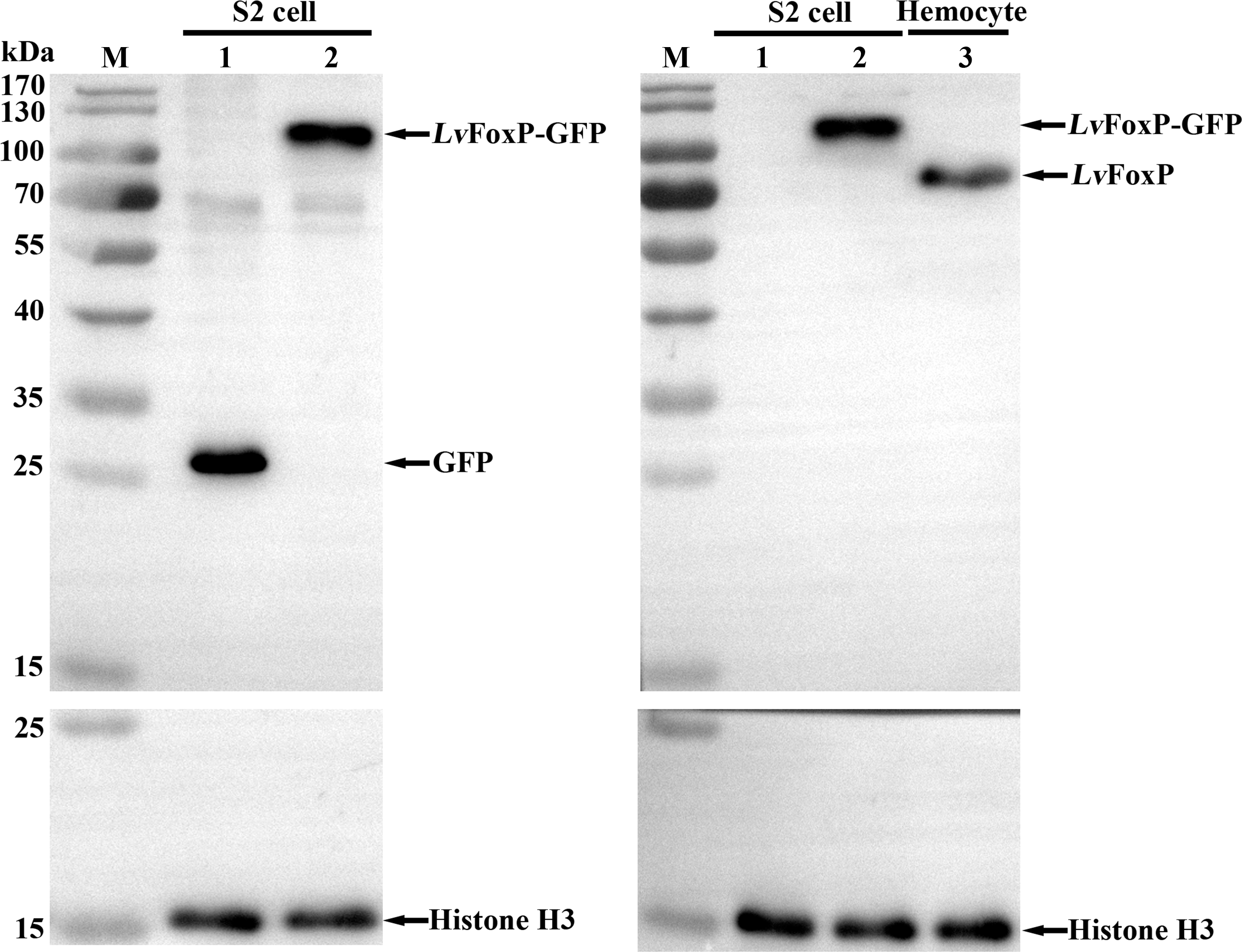

Supplement: Supplementary Figure 4 — Analysis of the specificity of the anti-LvFoxP antibody used in this study. Left panel: western-blot analysis of S2 cells expressing GFP (as control, line 1) and GFP-tagged LvFoxP (line 2) using anti-GFP antibody (Invitrogen, USA). Right panel: western-blot analysis of of S2 cells expressing GFP (line 1), GFP-tagged LvFoxP (line 2), and shrimp hemocytes (line 3, expressing natural LvFoxP) using anti-LvFoxP antibody. [file Image_4.tif]
