# Supplementary material for: A Novel Forkhead Box Protein P (FoxP) From Litopenaeus vannamei Plays a Positive Role in Immune Response
Source: Front Immunol. 2020 Dec 14;11:593987. doi: 10.3389/fimmu.2020.593987 (PMC7768020; doi:10.3389/fimmu.2020.593987)
Supplement: Supplementary file 5 [file Table_1.docx]

**Table S1 Sequences of primers used in this study**

|  | | | |
| --- | --- | --- | --- |
| **name** | **Sequence(5'-3')** |  |  |
| **spliced and verified** |  | **Real-time RT-PCR** | |
| *LvFoxP-1F* | CGAGCTCATGGCGCAGATGCCGCCCGT | *c-JNK-Q-F* | CCGCTACCCTGGCTATTCCTT |
| *LvFoxP-1R* | CCCTCGAGCTAGCCTTCT AGAGGAGGCCCTTGCC | *c-JNK-Q-R* | TCGTGCTTGACTTGCTTTGAG |
| *RACE* |  | *cMnSOD-Q-F* | TTGCCGCTACGAAGAAGTTG |
| *LvFoxP-3'RACE-A* | TGCCATCTTCCTCAACAAGCCAGC | *cMnSOD-Q-R* | AGAAGATGGTGTGGTTCAAGTG |
| *LvFoxP-3'RACE-B* | GGCTTGTGTACATGATGCAGGGAAACC | *PPO1-Q-F* | TCTTCGCCTCACGCATCTC |
| *LvFoxP-3'RACE-C* | GGCAATCATAGAGTCACCAGACAAGCAAC | *PPO1-Q-R* | TATCCTCACAGTCACCTCCTTC |
| *LvFoxP-5'RACE-A* | TGGCGGACGGCATTCTTCCAAGTTG | *PPO2-Q-F* | TCACGAACGCCGAGGAAC |
| *LvFoxP-5'RACE-B* | GTGTTCCTATTGCACCAAACAAAGACACG | *PPO2-Q-R* | GCAGCCGCAGAAGTTGAAAC |
| *LvFoxP-5'RACE-C* | AGGATTCCGCCAGGACTCTGCGAACG | *PPOAE1-Q-F* | CATCGTAGGCGGAAAGGAC |
| *Protein expression* |  | *PPOAE1-Q-R* | AGAATGTGACTGTCGGTGATG |
| *LvFoxP-KpnI-F* | GGGGTACCATGGCGCAGATGCCGCCCGT | *PPOAE2-Q-F* | AGTCGTCCTTCATCCTTCCTTC |
| *LvFoxP-XhoI-R* | CCCTCGAGGCCTTCTAGAGGAGGCCCTTGCC | *PPOAE2-Q-R* | GGATTGTGCGTTCCTGTTCAG |
| **dsRNA templates amplification** | | *Lys-Q-F* | CGGACTACGGCATCTTCCAG |
| *dsRNA-LvFoxP-T7F* | GGATCCTAATACGACTCACTATAGGA CAGGCAATCATAGAGTCACCAGACG | *Lys-Q-R* | TCATCGGACATCAGATCGGAAC |
| *dsRNA-LvFoxP-R* | CCTCCTCATGACGTGTCATTGCCTC | *ALF1-Q-F* | GGATGTGGTGTCCTGGATGG |
| *dsRNA-LvFoxP-F* | ACAGGCAATCATAGAGTCACCAGACGAG | *ALF1-Q-F* | GCGTCGTCCTCCGTGATG |
| *dsRNA-LvFoxP-T7R* | GGATCCTAATACGACTCACTATAGG CCTCCTCATGACGTGTCATTGCCTC | *ALF2-Q-F* | GCGAACAAACTCACTGGACTG |
| *dsRNA-eGFP-T7F* | GGATCCTAATACGACTCACTATA GGACGGCAAGCTGACCCTGAAG | *ALF2-Q-F* | ACATGCGACCCTGGAATACAG |
| *dsRNA-eGFP-R* | GACTGGGTGCTCAGGTAGTGG | *ALF3-Q-F* | GACCTGTCCAACCCTGAGC |
| *dsRNA-eGFP-F* | ACGGCAAGCTGACCCTGAAG | *ALF3-Q-F* | TCGCCTCCTCCTCCGTTATC |
| *dsRNA-eGFP-T7R* | GGATCCTAATACGACTCACTATA GGGACTGGGTGCTCAGGTAGTGG | *ALF4-Q-F* | CCTGGTGGCACTCTTCGC |
| **Real-time RT-PCR** |  | *ALF4-Q-F* | ACGGTGAAGCGGCACTTATG |
| *LvEF-1α-Q-F* | CCTATGTGCGTGGAGACCTTC | *LvALF-AvK-Q-F* | GTTCCTGGTGGCACTCTTCG |
| *LvEF-1α-Q-R* | GCCAGATTGATCCTTCTTGTTGAC | *LvALF-AvK-Q-R* | TCCGTCTCCTCGTTCCTCC |
| *LvFoxP-Q-F* | CCTGTATCTCACTACCGTCATTATTCT | *PEN2-Q-F* | CCAAGGCGAAGCGTACAG |
| *LvFoxP-Q-R* | CCTCATGTGAGCCTCGTGTAGTAAT | *PEN2-Q-R* | CAATTGCGAGCATCTGAGAC |
| *LARK-Q-F* | TGGAAAGCCTATGGTTGTTGA | *PEN3-Q-F* | CTCCTGCGTCCGCCATG |
| *LARK-Q-R* | GGAGCCATACACCTCAAATAGACT | *PEN3-Q-R* | GTGTAACCGCCCTTGTACAC |
| *MyD88-Q-F* | GGCAAAGGGCTATTGGAACTAT | *PEN4-Q-F* | GCCCGTTACCCAAACCATC |
| *MyD88-Q-R* | ATGATCCAGACACCTCTCGTATTC | *PEN4-Q-R* | AACAATCCCCGTATCTGAAGC |
| *IKKβ-Q-F* | ACCACACTTTCCACCTTTGG | *Cru-Q-F* | CACAACCTGTTCCAACGACTAC |
| *IKKβ-Q-R* | TCCCGATGAAGGAAGAACAC | *Cru-Q-R* | ACCTGCGATCCGAAGAATGAG |
| *IKKε-Q-F* | TTGGCTTCTTTCCAGGACAC | *Cru1-Q-F* | GTAGGTGTTGGTGGTGGTTTC |
| *IKKε-Q-R* | TTTTATGGCTGCCAGGAGTC | *Cru1-Q-R* | CTCGCAGCAGTAGGCTTGAC |
| *Dorsal-Q-F* | TTGCGACCACCAGACAAGAG | *CruA-Q-F* | CTTCCGACCTCCCTTCAACC |
| *Dorsal-Q-R* | GCAAGGTAACGACTAATCTTCTCTG | *CruA-Q-R* | CGTTGAAGCAAACCTGAGGC |
| *Relish-Q-F* | CTGCTTCTCCATACTCAGACCAC | *SWD3-Q-F* | CAGGAAGGTGCCGTGATGT |
| *Relish-Q-R* | CTGTGGCTGCTCCAGTATTTG | *SWD3-Q-R* | GGACAGCACTTGTAGCCGTATT |
| *STAT-Q-F* | CTTGCTCCGTTTCTCCGACTC | *SWD4-Q-F* | ACGAGCACCGAACAGGAAG |
| *STAT-Q-R* | GGATGGCGAAGGCTTTACTG | *SWD4-Q-R* | CGCAAGATAACCCACCAGG |
| *Domeless-Q-F* | CGGGCACCTCGGAGAATGA | *SWD5-Q-F* | TGGTTCTAATGGCAGCAGTTG |
| *Domeless-Q-R* | TTGGACTCGGGTGGAAAGC | *SWD5-Q-R* | ATGCTCAGATGGGAGGGACA |
| *JAK-Q-F* | CAATGGGATGGTCTGGAGGAC | *CTL4-Q-F* | CTTGGACGCTTATGTCACCTAC |
| *JAK-Q-R* | CTAGCGACAGAGGGTTTAGCG | *CTL4-Q-R* | CATCCTTGCTCTTGATGTAGTCG |
| *ERK-Q-F* | TCAATCCTCACAAGCGCATCA | *Hem-Q-F* | CTCTACCAGTGCTTCGCCTAC |
| *ERK-Q-R* | TGGCAGGTCATCAAGTTCCATCT | *Hem-Q-R* | GTCCGTGTTGTCCTTGTTGTTG |
| *p38-Q-F* | GCAGTATTGGCAGAAGAAGCA | *FAS-Q-F* | TTCCATCGCCAGTCTTGTCAG |
| *p38-Q-R* | CAGGTGCCCCTATGCTAATG | *FAS-Q-R* | CCACCTTCACCTCGTAGTCAG |
| *MAPK14-Q-F* | GTCGGCTCGCAACTACATAC | *Lec-Q-F* | CTTGGACGCTTATGTCACCTAC |
| *MAPK14-Q-R* | CAGCATCTTCTCTAGTAGGTCTAC | *Lec-Q-R* | CATCCTTGCTCTTGATGTAGTCG |
| *c-Fos-Q-F* | CCATTACAGCTGTGGCTACGAGT | **Absolute real-time RT-PCR** | |
| *c-Fos-Q-R* | GGTCTGTTCGATGTTCCTCAAG | *WSSV32678-F* | TGTTTTCTGTATGTAATGCGTGTAGGT |
| *c-JUN-Q-F* | CCATCCTCAACAGCAACACGG | *WSSV32753-Q-R* | CCCACTCCATGGCCTTCA |
| *c-JUN-Q-R* | CTGTTCCAAAGTGTCCTCAAATCCT | *TaqMan probe -WSSV32706* | CAAGTACCCAGGCCCAGTGTCATACGTT |
